# Leeches

**Published:** 1869-05

**Authors:** 


Leeches.—Leeches belong to a group of articulate animals of a division of the red-blooded worm. They have ten eyes around the lips. These eyes are very brilliant, and arranged in the form of a horse-shoe. They have three jaws, forming the three sides of a triangle. Each jaw is armed with teeth capable of piercing the toughest skin. The body of the medicinal leech consists of ninety-six to one hundred ringlets. As they grow the rings increase in size, but not in number. Their colors vary exceedingly, the most brilliant being that of the Australian leech. From official statistics it is found that the value of the leech sold in various parts of Europe, for use in home and foreign markets, aggregates the sum of ten millions of dollars annually, which amount is progressing from the diminished production in those countries themselves, and the consequent increased demand for leeches brought from Australia and other parts of the world. They are transported in clean linen sacks, or packed in clay or earth. Large numbers are lost by heat or confinement.—*Chem. Gazette.*

